# Exploring the Experiences and Well-Being of Australian Rio Olympians During the Post-Olympic Phase: A Qualitative Study

**DOI:** 10.3389/fpsyg.2021.685322

**Published:** 2021-05-26

**Authors:** Andrew Bennie, Courtney C. Walton, Donna O’Connor, Lauren Fitzsimons, Thomas Hammond

**Affiliations:** ^1^School of Health Sciences, Western Sydney University, Sydney, NSW, Australia; ^2^Elite Sport and Mental Health, Orygen, Melbourne, VIC, Australia; ^3^The Centre for Youth Mental Health, The University of Melbourne, Melbourne, VIC, Australia; ^4^Faculty of Arts and Social Sciences, The University of Sydney, Sydney, NSW, Australia; ^5^New South Wales Institute of Sport, Sydney, NSW, Australia; ^6^Ormond College, The University of Melbourne, Melbourne, VIC, Australia; ^7^School of Psychology, Deakin University, Melbourne, VIC, Australia

**Keywords:** Australian Olympic athletes, post-Olympic Games, mental health, qualitative research, interviews, well-being

## Abstract

Research about the Olympic Games has primarily focused on preparing athletes for competition. Less attention has been paid to the post-Olympic-phase (POP) and athlete well-being during this time. This study explored Australian Olympic athletes’ experiences following the conclusion of the 2016 Rio Olympic Games, including the factors that may have contributed to or challenged their well-being during this time. Eighteen athletes participated in semi-structured interviews and thematic analysis revealed that when Olympic performance appraisal met prior expectations, when athletes planned for a return to work or study, and when support from a variety of sources was readily available, this positively influenced athletes’ well-being during the POP. When these factors were not in place, more challenging post-Games experiences were present, and well-being was compromised. The findings contribute to the broader literature on elite athlete well-being and at an applied level, may be used to inform targeted programs that focus on supporting athletes after an Olympic campaign.

## Introduction

The Olympic Games are a large-scale sporting event that often reflect the pinnacle of an athlete’s career (Wylleman et al., 2012; Jensen et al., 2014). Competing in the Olympics can be associated with highly positive experiences such as national and international recognition, fulfillment of life-long goals, and attainment of financial benefit (Wylleman et al., 2012). However, as the spotlight fades on an Olympic campaign, athletes can experience unexpected challenges related to the post-Games phase that may lead to mental health issues which require coping processes (Schinke et al., 2015; Howells and Lucassen, 2018). Concern for elite athletes’ mental health has increased in recent years and new guidelines have been produced for supporting athlete wellbeing (Schinke et al., 2018; Henriksen et al., 2019, 2020; Reardon et al., 2019; Stambulova et al., 2020). However, limited information is available to understand the contextual challenges that athletes face, and what resources they draw upon to cope during the post-Olympic phase (POP). Therefore, further research is required to better appreciate athlete experiences during this period of the quadrennial cycle. Our exploratory study focused on the experiences of 18 Australian Rio Olympians throughout the POP, while also inquiring into the factors that contributed to or challenged their wellbeing during this time.

The Centre for Disease Prevention and Control (CDC) defines wellbeing as the presence of positive emotions and moods alongside the absence of negative emotions whereby life is judged positively in a range of psychological, social, economic, and physical domains (Centre for Disease Prevention and Control [CDPC], n.d.). Connected to wellbeing are definitions of mental health, which the World Health Organization classifies “a state of wellbeing in which every individual realizes his or her own abilities, can cope with the normal stresses of life, can work productively, and is able to make a contribution to her or his community” (World Health Organization, 2018, para 2). This definition was cited in a recent commentary on mental health research in elite sport (Poucher et al., 2021), while Kuettel and Larsen (2020) narrowed the focus of the WHO definition to describe mental health in elite sporting contexts as:

… a dynamic state of well-being in which athletes can realize their potential, see a purpose and meaning in sport and life, experience trusting personal relationships, cope with common life stressors and the specific stressors in elite sport, and are able to act autonomously according to their values (p. 253).

This contextualization is important because elite athletes are prone to unique stressors associated with sporting participation like injury, travel, tense coach-athlete relationships, and poor performance outcomes which can negatively impact on their mental health (Greenleaf et al., 2001; Poucher et al., 2018, 2021; Kuettel and Larsen, 2020). To understand Olympic athlete experiences during the acute period immediately following an Olympic event, it is imperative to first consider how performance expectations and outcomes influence athlete wellbeing through this time. This is because the competition event is the main focus for an athlete during an Olympic campaign and there is a significant relationship between competition outcome and emotional state (Hassmén and Blomstrand, 1995; Wilson and Kerr, 1999).

Researchers note that successful performances are typically associated with pleasant affect, increased motivation, improved self-confidence, and positive media attention during the POP (Wylleman et al., 2012). However, following an unsuccessful performance, the opposite trend is observed with evidence suggesting that more severe psychological consequences (such as post-Olympic depression) can emerge over time (Gahwiler, 2007; Gordin and Henschen, 2012; Hammond et al., 2013; McArdle et al., 2014; Samuel et al., 2016; Howells and Lucassen, 2018). Howells and Lucassen (2018) explored the concept of ‘post-Olympic blues’ with four female British Olympians who competed at the 2016 Games. Through retrospective interviews, they found that failing to meet pre-determined performance expectations led to a variety of negative affective states including anxiety, crying, and interpersonal hypersensitivity. Compounding these feelings was the sudden loss of ‘celebrity’ status (i.e., reduced media attention) and family members who did not fully understand the ups and downs associated with the conclusion of an Olympic campaign (Howells and Lucassen, 2018). While Howells and Lucassen detailed some impacts of performance expectations/outcomes on post-Games affect, their narrow sample of female athletes who did not win a medal means there is an opportunity to build on these initial insights with a broader range of athletes.

Upon departing an Olympics, athletes begin ‘The Homecoming’ (Howells and Lucassen, 2018, p. 71) and several studies have investigated factors influencing athlete wellbeing during this phase (see Gulliver et al., 2012; Wylleman et al., 2012; McArdle et al., 2014; Samuel et al., 2016). For instance, Wylleman et al. (2012) uncovered how contact with parents, family, partners and peers should increase, while support from a sport psychologist could also be considered to benefit wellbeing during this time. The four male athletes’ in Wylleman et al. (2012) suggested that formal workshops led by former Olympians in the months leading into an Olympic event could create a supportive environment in which to share other life-interests and ways to cope during the POP. Building on this concept of using formal programs to support elite athletes’ wellbeing following a Games event, McArdle et al. (2014) evaluated the impact of a post-Games program on 10 Irish Olympic and Paralympic athletes who competed at London 2012. They showed how athletes embraced many elements of the program for supporting wellbeing; however, athletes’ opinions differed when considering structural implementation factors such as the timing of mental check-ups and psychological debriefs. For instance, while there was relative consensus that a follow-up debrief was useful 4–5 weeks following the Games, debriefing immediately following or at the Olympics received “mixed reviews” (p. 276) given the considerably overwhelming nature of the event. Furthermore, McArdle et al. (2014) noted several barriers to program participation such as perceived stigma, low expectations, and inaccessibility (due to the regular centralized location). One of the key recommendations emerging from this study was the need for preparatory psychological education *before* the Games event so as to help athletes best cope with the *future* critical events. In sum, it appears that sport organizations hold significant responsibility for providing the formal structures and resources to support elite athletes, to cultivate their health seeking behaviors, and to lead educational initiatives pertaining to mental health literacy within and beyond the Olympic context (Henriksen et al., 2019; Gorczynski et al., 2020).

As time progresses during the POP, athletes begin to consider short and long-term career goals that impact on their wellbeing, such as returning to competition or retiring from sport. Athletic retirement has featured regularly in academic research, with a systematic review of athletes’ career transitions featuring 126 studies over a 40-year period (Park et al., 2013). Park et al. (2013) found that certain factors influence a more or less successful transition into retirement, including the level of autonomy in decision making about retirement, and the perceived success they achieved throughout their career. In some cases, post-Olympic career uncertainty—whether an athlete plans to retire or begin a new cycle of training for the next Games—has contributed to negative affect, making the transition to retirement a very challenging experience (Howells and Lucassen, 2018). Furthermore, Torregrosa et al. (2015) note that the strength of an athlete’s sporting identity [i.e., the degree to which an individual thinks, feels, and identifies with the athlete’s role (Brewer et al., 1993)] further enhances the difficulty experienced in career transition. However, it is currently unclear as to what role, if any, that career uncertainty plays in an athlete’s immediate POP, potential return to competition (i.e., for athletes who are not retiring), and impact on athlete wellbeing. To better understand how athletes navigated the transition to continued participation or retirement and its impact on wellbeing during the POP, further research is required.

There is a broad range of literature underpinning research into elite athlete wellbeing and sport transitions frameworks have often been used to underpin research in this field (Wylleman and Lavallee, 2004). Stambulova and Wylleman (2014) defined transitions as ‘… turning phases or shifts in athletes’ development associated with a set of specific demands that athletes have to cope with in order to continue successfully in sport and/or other spheres of their life’ (p. 609). The Holistic Athletic Career Model (Wylleman and Lavallee, 2004; Wylleman, 2019) provides one of the earliest frameworks to exemplify the connection between athletic (e.g., physical), psychological (e.g., motivation), psychosocial (e.g., family), academic/vocational (e.g., student/athlete), financial (e.g., sport governing body funding), and legal (minor/adult) factors influencing athletes’ transition experiences at various levels of development throughout their athletic and non-athletic careers. The Athletic Career Transition Model (Stambulova 2003, cited in Stambulova and Wylleman, 2014, p.10) focuses on the various barriers (e.g., low self-efficacy), resources (e.g., previous athletic and personal experiences), and coping strategies (e.g., planning, social and professional support) related to athletes’ transition demands during their careers within and beyond sporting contexts. More recently, Samuel et al. (2020) combined various components of earlier transition models to generate the Integrated Career Change and Transition Framework. This model captures the (a) career change event; (b) transition demands; (c) the athlete’s appraisal of transition demands, resources, and barriers; (d) the athlete’s strategic decision making in relation to how to cope with a career change event; and (e) positive or negative transition outcomes. Specific to the Olympic context, Schinke et al. (2015) viewed the Olympic Cycle to comprise of several transition phases from pre-Olympic (National Team Selection, competing at international tournaments, Olympic qualification, and preparation for the Games) to in-Games participation, and the post-Games phase. A 2012 qualitative study with four male Beijing Olympians confirmed that athletes experienced a range of changes through these transitory phases in athletic, psychological, psychosocial, and academic vocational domains, demonstrating the potential utility of such frameworks for research exploring elite athletes transition experiences (Wylleman et al., 2012). However, these frameworks have only intermittently been implemented as a framework for research within the post-Olympic setting (e.g., Wylleman et al., 2012) and even less within southern hemisphere contexts.

As a result, we chose to draw upon these models in conjunction with previous literature as a lens from which to understand athlete experiences during the POP rather than strict theoretical framework for shaping the research, interpreting data, or discussing findings. More specifically, we preferred to be open minded about investigating the topic given the limited attention that national Olympic committees invest in the post-Games period (Henriksen et al., 2020) and the fact that few studies have given voice to athletes about what happens during this time. Therefore, the purpose of this study was to investigate what Australian Olympic athletes experienced following the conclusion of the 2016 Games. Our aim was to explore what, how, and why certain factors contributed to or challenged athlete wellbeing—as well as the strategies athletes used to cope—during the POP.

## Materials and Methods

As the focus of this study was to understand Australian athletes’ unique experiences in the specific context of the 2016 post-Olympic period, the current project employed a qualitative design based on a post-positivist philosophy (Poucher et al., 2019). This philosophical stance seeks to accumulate knowledge in the pursuit of reality, but also acknowledges that the ‘truth’ for one person may not be the same as for others (Coulter et al., 2016; Poucher et al., 2019). Ontologically, this assumes that one universal truth “may never be fully understood” given the complexity of subjective human experience, restrictions of human language, and limiting methodological tools. Epistemologically, the goal is to minimize influence of the researcher on the researched in an effort to “produce knowledge that is as objective as possible” (Poucher et al., 2019, Supplementary Materials, p. 2) while gaining a better grasp of what reality might be in the specific contexts under investigation (Coulter et al., 2016). Hence, we approached this investigation with a belief that there could be ‘universal POP experience of Olympic athletes’ (although we may never completely understand it).

### The Australian Olympic Context

In 2016, Australia sent a team of 422 athletes and finished eighth on the medal tally – well below expectations (Australian Olympic Committee [AOC], 2016a). Throughout the quadrennial cycle, athlete support remains the responsibility of Australia’s National Institute Network and National Sporting Organizations (NSO’s), with the exception of the pre-Games period from Olympic selection through to the Closing Ceremony. Amongst the AOC’s key objectives is a commitment to support the overall health care of athletes; however, their remit to deliver athlete support only formally commences once an athlete is selected and signs the Australian Olympic Team Membership Agreement (Australian Olympic Committee [AOC], 2016b). Following an Olympic campaign, athletes are offered access to an extended network of athlete wellbeing and psychological support services under the Elite Athlete Brief Counselling Support Program (Australian Sports Commission [ASC], 2016). Athletes are also advised to seek any required post-Games support from coaches, support staff; and/or via the support services provided by their NSO and home institute of sport (Australian Sports Commission [ASC], 2016). However, athlete support is impacted by various sport-specific nuances, including available resources, historical Olympic performances, and the size of the athlete cohort. For example, Foundation sports (those with a high probability of achieving a gold medal) such as swimming and sailing are prioritized under the sport categorization framework, and funded accordingly (Australian Institute of Sport [AIS], 2018).

### Sampling, Recruitment, and Participants

In early 2018, the AOC commissioned our research team to carry out the present research^1^. Following university ethics approval (#HEAG-H 45_2018), athletes were purposively (Braun and Clarke, 2013) sampled from the 2016 Australian Olympic team so as to learn directly from their experiences during the POP. Although nearly 2 years after the Games, Howells and Lucassen (2018) explained that conducting research after a significant period of time allows adequate time and space for athletes to adjust, reduces heightened emotions often associated with intense sporting performances, and enables them to reflect upon, and reframe their initial perspectives of the event. These were important considerations given that some athletes may suffer from retrospective bias or be heavily influenced by current thinking/life experience at the time of the interview.

Because so few studies have been conducted with a focus on the POP, we sought a diverse range of Olympic athletes rather than selecting a sample from a specific sport, gender, medals won, or number of prior Olympic experiences. As such, the final sample relied on a convenience sampling approach (Patton, 2015). To recruit athletes, staff from the AOC distributed the study advertisement among their database of Rio Olympians. Athletes then either contacted the research team directly or provided their details to the AOC, who then forwarded this information to the researchers. When arranging athlete interviews, all AOC personnel were removed from communications to ensure athletes’ anonymity. Out of the 422 athletes who were contacted to participate in this research, 18 agreed to be interviewed and were aged from 22 to 35 years (*M* = 28.33; see Table 1 for participant details). To protect athlete’s anonymity and reduce any identifiable features, pseudonyms were used, and their specific sports and competition outcomes were not included.

**TABLE 1:** Participants.

**Pseudonym**	**Sport**	**Sex**	**Age range at Rio**	**First time or multiple olympian**	**Retired or continued post-Rio**
Banhi	Team	Female	20–30	First	Continued
Bonnie	Individual	Female	30–40	First	Retired
Caitlin	Individual	Female	30–40	First	Continued
Hamish	Team	Male	30–40	Multi	Continued
Harper	Team	Male	30–40	Multi	Retired
Karen	Individual	Female	20–30	Multi	Continued
Katie	Team	Female	20–30	First	Continued
Kendrick	Team	Male	20–30	First	Continued
Rayanne	Team	Female	20–30	First	Continued
Sacha	Individual	Male	20–30	First	Continued
Saif	Team	Male	30–40	Multi	Continued
Shawn	Individual	Male	30–40	Multi	Continued
Swain	Individual	Male	30–40	First	Retired
Swana	Individual	Female	20–30	Multi	Retired
Swen	Individual	Male	20–30	Multi	Continued
Talia	Individual	Female	30–40	Multi	Continued
Triya	Individual	Female	30–40	Multi	Continued
Walid	Team	Male	30–40	Multi	Retired
Totals	Team (*n* = 8) Indiv. (*n* = 10)	Fem (*n* = 9) Male (*n* = 9)	20–30 (*n* = 11) 30–40 (*n* = 7)	First (*n* = 8) Multi (*n* = 10)	Cont. (*n* = 13) Retired (*n* = 5)

### Data Collection

Each participant took part in one semi-structured interview with one of two authors who were trained in qualitative research methods and have previous qualitative research experience^2^. The semi-structured interview guide enabled a flexible exploration of the athletes’ Olympic experiences and probing questions to obtain additional information of interest during the conversation. The interview started with a series of introductory questions about the athlete’s sporting experiences over their lifetime; general experiences leading into the Rio Games, and brief descriptions of experiences during the Rio Games. These questions were used primarily to establish rapport and familiarize the athletes with the interview process rather than to specifically address the research questions. As we were interested in learning about the immediate period of time following the completion of an athlete’s competition event (i.e., still in Rio) right up to how they were feeling at the time of interview, athletes were then asked about their post-Olympic experiences and factors that may have contributed to, or challenged, their wellbeing during this time. As such, key questions focused on what happened, the challenges faced, and what resources athletes drew upon to cope during the POP. Because definitions of post-Olympic timeframes varied in past research with Olympic athletes (e.g., Howells and Lucassen, 2018 interviewed athletes up-to 11 months after Games; McArdle et al., 2014 at least 3 months post-Games) and the fact that we conducted interviews nearly 2 years after Rio, we were open to athletes’ interpretations of what they considered the post-Olympic timeframe to include. As a result of the open time frame, the athlete’s articulation of when things happened, how, and why, emerged naturally during the interview. The interviews concluded by asking athletes to make recommendations for current and future Olympians, their coaches, families, and administrators to help improve experiences during future POP.

Depending on what was most convenient to each athlete, interviews were conducted in-person (*n* = 6) or via telephone (*n* = 12) and interviews ranged in length from 45 to 120 min (*M* = 60 min). These interviews were transcribed verbatim in preparation for data analysis and data collection ceased when no additional athletes volunteered their time to take part in the interview process.

### Data Analysis

After uploading transcripts to computer software program NVivo 12, data were analyzed following Braun et al. (2016) and Braun and Clarke (2019) guides to thematic data analysis techniques. This six-step process enabled the researchers to detect patterns within the transcripts when exploring athlete descriptions of the who, what, when, where and why of key events related to the POP (Poucher et al., 2018). First, individual transcripts were read multiple times to ensure familiarity with the data. Next, important data were identified inductively and coded semantically, whereby chunks of data (quotes) were labeled in a manner that closely reflected the athletes’ interview dialog (Braun et al., 2016; Braun and Clarke, 2019). Then, the preliminary codes were re-read to actively seek similar codes or differing insights before defining initial descriptive themes. Following this, two authors engaged in a critical dialog (Smith and McGannon, 2018) to review the coding and thematic processes. Here, interpretations of the data were discussed to explore the researchers’ subjective analytic insights and develop a more collaborative and nuanced understanding of the data before finalizing the thematic descriptors and underlying content (Braun and Clarke, 2019). The final analytic step involved writing up the data based on the overarching themes, underlying concepts, and supporting quotes.

### Quality Standards

When reflecting on qualitative research, it is important to consider the quality of the process. In this section, several of Smith et al. (2014) quality standards for judging qualitative research (italicized below) are addressed. In the results section, a diverse range of quotes are provided so as to enhance the *width* and comprehensiveness of specific findings, and, when combined with the explanatory notes surrounding key quotes and themes, this serves to present a *coherent* narrative of the key events that our sample of athletes voiced in relation to the research topic. Given the limited attention the POP has received from the academic community, this is *worthy topic* for consideration. The results are underpinned by a *sincere* and *credible* approach (Smith et al., 2014) to build on existing research where a level of critical dialog was maintained with athletes (e.g., feedback on interview transcripts) and analysis with fellow authors (e.g., about interpretations of data) during the various stages of the research project (Smith and McGannon, 2018).

## Results

**FIGURE 1:**
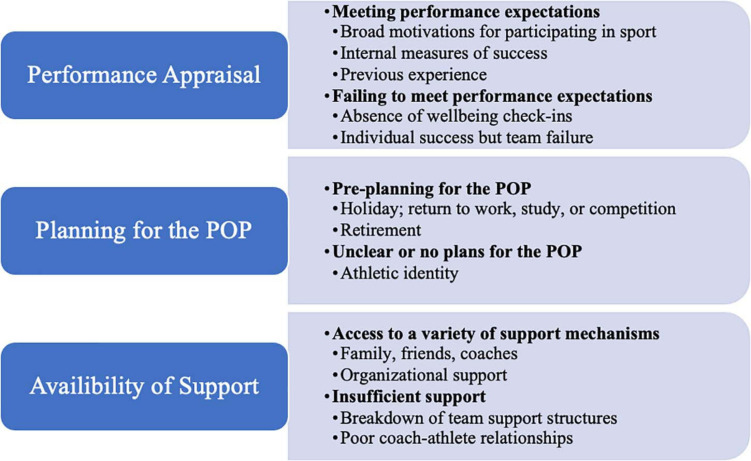
Visual representation of themes.

The findings from this study highlight the core post-Olympic experiences of a select group of Australian 2016 Olympians under three main themes: performance appraisal, planning for the POP, and availability of support. The results describe the positive and challenging experiences of the athletes under each thematic heading to give an indication of what contributed to their wellbeing and capacity to cope well (nor not cope well) as they navigated the POP. Quotes are used to exemplify the clearest examples of athlete experiences during the POP and although it may appear as though just a few athletes’ ideas were included in the below text, the themes and underlying messages represent the collective perspectives of athletes, unless stated otherwise. Figure 1 provides a visual representation of the findings.

### Performance Appraisal

When athletes appraised their Olympic performance in relation to prior expectation, they described whether or not they met performance expectations and how they responded to this appraisal during POP. Additionally, athletes often viewed their response to performance outcomes in fluid terms, meaning that some athletes described negative feelings in the immediate POP, but in the longer term spoke of their overall POP in positive terms. As such, it was not always possible to specifically describe athlete experiences in terms of coping or not coping well.

#### Meeting Performance Expectations

Regardless of their context (i.e., first-time or multi-Olympians, retiring or continuing etc.), when athletes met perceived performance expectations or were satisfied with their performance, they reported positive post-Olympic experiences and coped well during the POP. For instance, Swana’s athletic performance matched prior expectations, which was critical to her wellbeing as she progressed into retirement:

My ultimate goal was to just do a PB [personal best], because I hadn’t done a PB … for 5 years … I finally did my PB in the final, and honestly for me, doing a PB at that point in my career was like winning a gold medal. I know it’s a cliché, but I did really have a fairy tale end to my career.

Similarly, Shawn reflected upon on his broad motivations for participating in sport and previous experiences to feel positive about his Olympic performance:

I’m [in a] pretty good place with Rio … I mean it obviously depends on … what you’re doing the sport for … There are so many athletes that are doing it because they need validation from outside source … with Rio I was a bit older and didn’t really care if people liked me or not. So then I was able to do it more for myself and it didn’t matter what happened afterward. If I was happy with my performance, then that was enough.

Athletes like Shawn and Swana focused more on internal expectations rather than how others perceived their performance, and these internal measures of success were critical for responding positively to their Olympic performance and coping well during the longer term POP.

#### Failing to Meet Performance Expectations

Unfortunately, most of the athletes in this sample perceived that their performance failed to meet expectations (i.e., they did not set a personal best, make a final, or win gold) or were disappointed with their Olympic performance outcome. These adverse perceptions negatively influenced athletes during the immediate POP and for some athletes, led to negative and longer-term psychological distress. Hamish’s comments provide important insights for those seeking to assist athletes overcome negative performance appraisals when navigating the POP, as it appears crucial to check in on their wellbeing well beyond the Olympic Games:

I reckon I got over Rio, or not got over, but I started to let go of Rio, when I got close to the Comm Games [Commonwealth Games in 2018] because I thought to myself, I just don’t want to be thinking about Rio when the Comm Games is in my backyard. So, I reckon … we’re talking 14–15 months after Rio, I let go of Rio in some ways.

Further demonstrating the complexity of how athletes appraise their performance, retiring athlete Walid acknowledged that while his team failed to meet their performance expectations, he was satisfied with his individual performance:

I had probably my best international tournament in Rio, but it counted for nothing cause we lost. So it’s a tough balance, can I come back proud of how I went individually? Well yeah but I’ll probably think about it in 20 years like having to explain why we didn’t at least fight for a medal.

These conflicting personal feelings alongside the perceived pressure he felt with this being his final Olympic campaign, challenged his self-perception during the POP. They also demonstrate that differences may occur between team and individual athletes when responding to performance expectations, which is important for planning athlete support during this time. In sum, a range of different responses emerged when athletes appraised their performance in relation to expectations, confirming a strong connection between their performance outcome, athletic identity, and wellbeing during the POP.

### Planning for the POP

All athletes described how planning for the POP prior to the Olympics served as a protective factor for their wellbeing while navigating the POP. Alternatively, failing to plan for the POP or being uncertain about retirement instigated more negative feelings during this time.

#### Pre-planning for the POP

Many athletes who shared positive experiences during the POP had pre-planned something to do for the POP. For these athletes, simply having a plan appeared to facilitate “a positive mindset because you’ve got these fun, exciting, awesome things to look forward to” (Shawn). This demanded a clear post-Olympic plan *prior to* Rio where some athletes scheduled a holiday, returned to the comfort of their home, and/or returned to regular sporting competitions. For Bahni, the physical and mental benefits of her returning to compete soon after her first Olympic Games were clear:

… my [professional league] season … started within 10 days after [Rio]. I actually kind of found it refreshing because I could refocus. And I distinctly remember being on a road trip and talking about what happened in Rio with the girls and stuff and kind of being able to debrief and get my frustrations out and that with the coach as well as the players in our team.

While returning to competition had a positive effect for certain athletes, others felt that time away from sport and the team environment was a necessary break from the hype of the Olympics and helped them cope during the POP. Planning a return to work or academic studies was another protective factor drawn upon during the POP. First-time Olympian Kati described how having a strong connection to work and study (i.e., things beyond her athletic identity) were integral to coping well during her POP:

I just love being in my routine at home. I have a lot going on outside of [sport removed], and I don’t define myself as a [sport removed], we own a business, and I’m finishing my MBA soon, and there’s lots of other things going on. So I really love being at home and being able to immerse myself in that. When I’m overseas and traveling and racing its [sport removed], and I love it, but I need to have other things in my life.

Overall, after being exposed to an emotionally charged, highly stressful, and exciting major life event presented athletes with a series of emotions and academic/vocational demands to contend with. For instance, while all athletes experienced an immediate sense of relief following the completion of their event, many athletes described a short-term emotional ‘come down’ (upon leaving the Games, and in most cases returning home) that often presented in negative physical and psychological symptoms upon returning home. Kendrick’s explained how this unfolded for him during the POP:

… it’s just the pressure release … that following week, I was pretty Olympic’d out and … definitely that first 2 weeks … I was just really, really tired and … super, super run down straight after the Games … I think that was just mainly this huge stress relief sort of thing.

Even though Kendrick described his immediate POP feelings as a barrier to coping well, he spoke at length about drawing on psychosocial and vocational resources—like being back home, returning to work, and planning time with family and friends—as a mechanism for coping positively as time progressed during the POP:

I just wanted to relax and, yeah, just hang out at home and, which I did … And then I thought I could end up going surfing for 2 months just at home. And, what else did I do? I renovated the kitchen, that was pretty fun. But besides that, I was pretty sweet. I just sort of got straight back into work. Luckily, I really like my job so that always helps. And, yeah, just started sort of working on the house and stuff and bought a dog, that was cool.

Therefore, acute responses during the POP did not necessarily translate into longer term challenges when adequate plans to return to ‘normality’ were made prior to the games.

#### Unclear or no Plans for the POP

When athletes did not have clear plans for the POP—like being uncertain about whether to retire or continue competing—this generated negative emotions. Walid explains the stress induced under such circumstances as he wrestled with his strong athletic identity and the reality of what might happen in his post-athletic career during the immediate and longer term POP:

I was a bit stressed because I didn’t have as much direction as I sort of planned. I thought it’d be very clear after Rio what I was going to do, and it wasn’t … I think the hardest thing was realigning my goals … When you start a new career and you’re starting at the bottom rung, it’s sort of like, how do I move that set of goals which have been so clear for me to become the best in the world and put it somewhere else? And that might be, I want to be a great dad, or that might be, I want to be great at community service, or something like that. But it’s not a clear, tangible thing that, as an athlete, you genuinely believe that you can … achieve something that other people can’t.

Challenging experiences did not appear to be different for certain multi-Olympians even though they may have been able to draw on previous Olympic campaigns to manage their POP wellbeing. For instance, Swen outlined how social isolation in the immediate period following the Games served as a barrier to effective coping:

When you get home it’s really lonely; you miss hanging out with all your friends and waking up with them, and walking and hanging out with them, so there is an element of loneliness. It’s quite depressing, and it is a little bit overwhelming, starting from square one again.

Overall, planning a returning back to normality or time away from the sport following an Olympic campaign played a critical role in athlete wellbeing during the POP.

### Availability of Support

Athletes described how the availability of psychosocial and financial support (or lack thereof) from family, friends, coaching staff, and sporting organizations critically influenced their wellbeing during the POP. More specifically, athletes with strong support networks reported more positive post-Olympic wellbeing; whereas those who did not or could not access support struggled. Furthermore, national system stressors including organizational restructures, coaching changes, and funding cuts were clear impediments to athlete wellbeing during the POP although support from these entities often provided a necessary buffer during this time.

#### Access to a Variety of Support Mechanisms

When recounting the time immediately following competition, athletes noted how informal debriefs with coaches, subsequent celebrations with family members, and staying on after their event to support teammates positively impacted on their wellbeing during the POP. Various athletes also explained how connecting with friends outside of sport was beneficial and Kendrick explains how this process provided the necessary buffer to distract from the hype of the Olympic event and generate feelings of normality:

… I reckon that’s massively important, just having people and people who aren’t involved in sport at all. They’re the sort of people who … if you win it’s all … like “oh, yeah, that’s awesome” and if … you don’t win, … they just don’t really care about it, which is great because it gives you that mental break.

Alternatively, other athletes suggested that reconnecting with friends from within their sporting circles soon after returning home from Games was useful because they could understand what they were going through. While certain athletes suggested informal get togethers helped them feel socially connected in the months following the Games, they also mentioned the value of formal mentoring programs or internship opportunities (regardless of career stage) with past Olympians or the broader working community. These informal and formal arrangements helped create networks to draw upon for support and career development and positively impacted on athlete wellbeing during the POP. Overall, athletes highlighted their individualized and varied preferences for engaging family, friends, and other networks to help them cope during the POP.

Athletes who returned home immediately following the Games were able to attend celebration parades across various parts of Australia. While these public forms of support generated feelings of excitement, Rayanne explained how her team’s unexpected success and ensuing ‘celebrity of winning’ raised a number of unanticipated challenges during the POP:

We weren’t prepared for how big of an impact the [performance] would have at home, either; we didn’t realize the magnitude of how many young girls and boys or … how many people would message us on Instagram and Facebook and people wanting interviews, and media, and all that kind of stuff. We had no idea about the magnitude of how it would be.

This unique experience exemplifies the complicated and contextualized nature of how various experiences during and after the Games influence athlete wellbeing. Experiences like this also highlighted the importance of staying connected to athletes regardless of their performance outcome during the games. Indeed, when personal coaches regularly kept in touch (weekly/fortnightly) with athletes and initiated formal debriefs 1-to-3 months after the Games, athletes felt well supported. Additionally, when athletes received support via follow-up calls/meetings from Athlete and Career Educators (ACE) or psychologists from National Sporting Organizations (NSOs), this helped navigate some of the psychological challenges associated with their transition back to normality, as Talia explained:

At the [institute of sport] I had an ACE coordinator … she was good, she would check in probably every 6 months to see if I was doing okay, how my wellbeing and quality of life was and we’d have a little bit of a chat. She sent me off to do a meditation, a bit of yoga – anything out of the box, but that stuff helps – people checking in and sort of forcing you to talk about it … having that support network there to improve your emotional wellbeing.

These formal and informal touch points appeared to be critical resources for checking in on an athlete’s wellbeing, regardless of whether athletes were coping well or not during the POP. What remained unclear from the interview was whether athletes viewed their sports as having the necessary financial and human resources to execute the abovementioned strategies with enough regularity to really have an impact on the athlete’s capacity to cope during the POP.

#### Insufficient Support

Unfortunately, there were situations where athletes identified extremely challenging post-Games wellbeing, which tended to arise when psychosocial support networks disintegrated. For example, when teams were disbanded due to coaching and support staff contracts expiring (or being fired), or when fractious coach-athlete relationships arose due to unaligned perceptions of the athlete’s performance-that is, when athletes received poor feedback from coaches (or no communication) following what the athlete thought was a reasonable Olympic performance-athlete-coach relationships became strained, and reductions in athlete wellbeing was evident. Furthermore, uncertainty about whether or not funding was going to be continued during the POP contributed to another source of stress, particularly for female athletes from less well funded sports (e.g., combat sports). Bonnie lamented that situations like this were often compounded when NSOs did not offer support to fill the void:

So I had my head of my team, the strength and conditioning coach … He was the one who had helped me plan from 2015 how to make the Olympics … Then I had a psychologist, the national coach, [NSO removed], nutritionist, massage therapists, all that sort of stuff. Everyone was helping me … Post-Rio, I didn’t get contacted by a single person, not one of them contacted me … And I tried to contact them and there was no contact, there was no nothing … I lost all my funding. The Government just completely cut me. [Sport Governing Body] completely cut me… and I was just mentally fucked up. No assistance or help … I just got lost in the system.

As a first time Olympic athlete, Bonnie’s severe situation was unique amongst athletes in this study. However, there is scope for other athletes within the Australian Rio Olympic team of more than 400 to have shared this distressing experience. In the absence of receiving organizational support and in light of Bonnie’s feelings of abandonment, she then provided a novel solution for NSOs that may counter negative experiences like this in the future to better support athlete wellbeing during the POP:

So I would definitely create … an athlete liaison program … it might be as simple as they’re set up at the Olympics, maybe through the AOC or through the Sports Commission … and they just [say] here’s a card, when you’re ready post-Games, send us a text message and we’ll call you. Or set it up now and organize when you would like, 2, 3, 4, and 10 weeks, and we’ll give you a call … Just basic human contact I think is what people need … ‘Hey, how are you going? Do you need anything? Here are the resources available, let us know if you need them.’

Similarly, Triya suggested that the current gap in mental health service provision for athletes could also be addressed by adding “post-Olympic wellbeing seminars” into existing pre-Olympic information sessions (that the AOC already coordinates). Her sense was that these could be utilized as a resource to advise athletes about psychological strategies and services that could be employed during the POP if wellbeing is compromised. Additionally, several athletes at different career stages noted that it would be useful to seek input from athletes *prior to* the Games regarding type of support and time at which they might like to receive support during the POP.

While NSOs appear to have a significant responsibility to provide ongoing support for Olympians, some athletes recommended that it be left up to the individual to make the call about drawing on psychological resources during the POP. This form of individualized planning/support appeared particularly critical because as Triya explained, there is stigma associated with seeking mental health support among Olympians:

It’s that vulnerability of saying, “Yeah, I’m not coping,” I guess that is what keeps me just looking within my inner circle, because it’s not something you want to put your hand up and go, “Yeah, I’m over here. I’m struggling massively.” There’s still that stigma involved with it … (Triya).

While certain athletes reported positive psychological consequences as a result of engaging with sport psychologists during the POP, Triya mentioned that the high turnover and a lack of consistency of available psychologists during an Olympic cycle made it difficult to build trusting relationships. Hamish raised another barrier that impinged on his wellbeing during the POP, where independent psychologists were not available for consultation:

I would have absolutely spoken to a sports psych or a performance analyst or someone who wasn’t directly involved in my team. I felt it was so hard for me to speak to people within my own team because there was so much blame … I had some conversations with our sports psych, but I didn’t, I couldn’t talk about anything that I wanted to, because it was too close.

Overall, National system stressors and organizational change served as clear barriers to coping well during the POP.

## Discussion

The purpose of this study was to explore the experiences of 18 Australian Olympic athletes during the period immediately following the 2016 Games. The results revealed that performance appraisal, planning for the POP, and the availability of support influenced athletes’ wellbeing in positive and challenging ways as they navigated the POP. The findings contribute to the broader literature on elite athlete wellbeing and at an applied level, may help inform the development of targeted programs that focus on supporting athletes before, during, and after an Olympic campaign.

At the conclusion of their competition event, athletes at various career stages described how a sense of elation was often matched by fatigue. There were also challenges associated with the comedown from the Games matching the Howell’s and Lucassen description of “post-Olympic blues” (2018, p. 67). However, the most profound factor influencing athlete wellbeing during the immediate POP related to whether or not an athlete’s performance outcome met prior expectations, which reinforces previous research where athletes’ appraisal of actual performance in comparison with expectations played a critical role in their initial state of wellbeing (Hassmén and Blomstrand, 1995; Wilson and Kerr, 1999; Greenleaf et al., 2001). Importantly, when athletes in this study focused on their own performance rather than discreet or dichotomous outcomes (i.e., winning or losing) and the broader impact they had as an Olympian (i.e., like inspiring young people), this served as a critical internal strategy for coping more effectively during the longer-term POP. Alternatively, when athletes perceived they failed to meet performance expectations—which the majority of athletes in this study described—more challenging post-Olympic experiences emerged. What remained unclear, however, was whether the athlete’s performance objectives in the present study matched their sport organization and coach expectations (as they were not interviewed in this study). Whether self-imposed, or externally driven, expectations may have been unrealistic in some cases, particularly considering that less than 10% of athletes medal at Olympic events (Samuel et al., 2016). Taken together, maintaining realistic performance expectations appears critical to facilitating coping strategies (Stambulova, 2016), perceived performance satisfaction (Wylleman et al., 2012; Samuel et al., 2016), and a state of positive wellbeing following an Olympic Games.

Strongly influencing the athlete’s wellbeing during the POP were a range of psychological, social, academic, and vocational factors previously outlined in Wylleman’s Holistic Athlete Career Model (2019). For instance, some Australian Olympic athletes intentionally planned to detach from their athletic identity by returning to work or study while others celebrated being an Olympian in public parades. In fact, having something to do—a holiday or return to competition or work/study—was a specific resource used to cope, while those without a plan or who were uncertain about their future faced major barriers to wellbeing during the POP. Decreasing uncertainty in various aspects of Olympic preparation has been shown to help athletes stay confident while under pressure (Gould and Maynard, 2009; Stambulova et al., 2012). Furthermore, when athletes pursue interests and goals unrelated to their athletic careers (i.e., work/study), it can assist with coping and enhance their state of wellbeing after an Olympic campaign (Gordin and Henschen, 2012; Barker et al., 2014; Schinke et al., 2018; Kuettel and Larsen, 2020). Taken together, there is great value in supporting athletes with pre-planning their post-Games experiences *before* the Olympic Games to best support return to academic and vocational pursuits after competition finishes.

Our findings underlined the importance of psychosocial support from family, friends, and coaches, which has been well documented in previous research with elite athletes (Greenleaf et al., 2001; Brown et al., 2019; Teques et al., 2019; Samuel et al., 2020; Walton et al., 2021). Whilst most athletes indicated a strong sense of support from friends, family, and coaches, levels of organizational support were vastly different for all athletes in this study. When psychosocial support from coaches and governing bodies was absent, athletes were particularly vulnerable to negative post-Olympic wellbeing. In addition, coaching and support staff persistently tread the fine line of contractual termination at the end of an Olympic cycle and if relationships between athletes and coaches are soured by distrust (Greenleaf et al., 2001; Henriksen et al., 2020; Kuettel and Larsen, 2020), sporting organizations must consider where the ownership for athlete wellbeing should fall during this critical Olympic phase. Overall, these findings have practical implications for Olympic athletes and their National Sporting Organizations because sporting organizations must consider where the ownership for athlete wellbeing should fall during the POP

In Australia, National Sporting Organizations (NSO) recently affirmed athlete welfare as priority via increased investment in wellbeing and psychological support (Dunn, 2014; Australian Sports Commission [ASC], 2016; Sport Australia, 2019). For instance, the Australian Institute of Sport established a Mental Health Unit in 2018, where athletes were provided with access to appropriate practitioners through a Mental Health Referral Network (Rice et al., 2020). However, a lack of targeted programs for the POP may compound negative transitions (Gordin and Henschen, 2012) as athletes may feel vulnerable when reaching out for psychological support due to the stigma associated with help seeking behavior (Gulliver et al., 2012; McArdle et al., 2014; Poucher et al., 2021). To overcome this barrier, athletes in this study suggested that check-ins could be delivered via a neutral and centralized athlete wellbeing liaison officer-appointed pre-Games, by relevant NSOs-to provide individualized athlete support during the POP. Additionally, our findings echo Henriksen et al. (2020) recommendations that mental health screening and treatment be made available for athletes in the year following an Olympic cycle. Therefore, it appears vital for the governing bodies to step in and provide the necessary support when gaps in wellbeing service provision exist because the absence of follow-up could lead to problems for athletes who are hesitant to reach out, or, where relationships with team staff have broken down. However, sport psychology and mental health practitioners should take note that athletes in this study had mixed feelings about psychologists being engaged in the sporting system during the Olympics. Some athletes were very supportive of knowing the psychologist well, while others wanted to work with a psychologist completely disengaged from their sporting environment because of concerns with confidentiality. These distinct inclinations demonstrate the specific stressors that can arise in elite sport (Kuettel and Larsen, 2020), and so taking an individualized approach to planning psychological support appears vital so they can act according to athlete preferences during the POP.

### Limitations and Future Directions

Although conducting interviews approximately 2 years after the conclusion of the OG provided adequate time for athletes to adjust or reframe their perspectives (Howells and Lucassen, 2018), this could be considered a limitation of the study. Collection of data in closer proximity to the POP may enhance the accuracy of athlete perceptions by reducing the reliance on longer-term recall (Howells and Fletcher, 2015). As such, future researchers could consider carrying out multiple interviews with athletes or an ethnographic research approach to capture shifting perspectives over time as they navigate their journey back to normality. In addition, our study focused solely on the perspectives of athletes. It would be beneficial to purposefully sample the accounts of coaches, psychologists, and team managers within specific sporting case studies to gain a more holistic insights into the POP. While athletic retirement has been a popular topic for researchers, there is also an opportunity to more specifically investigate whether retirement was voluntary or not, and how these situations impact on the mental health of athletes during the POP. Echoing suggestions from Wylleman et al. (2012), utilizing retired Olympic athletes to mentor current athletes appears to be a good strategy for helping cope during the POP as they are well positioned to share in athletes’ experiences within (i.e., living in an Olympic village) and beyond the sporting context (i.e., how to cope after returning from the Olympics). However, further research is required to solidify how, when, where, and whom this form of support might be best placed to be delivered. Finally, future research could be more specifically framed by existing elite athlete transition models as they have yet to be tested for their applicability to Australian contexts and these models can provide guidelines about the context, time frame, methods, and type of supports practitioners can provide during the POP (Stambulova, 2016). Results from these studies could be utilized to improve assistance to athletes during this transition phase and address the current gap between research and practice (Stambulova et al., 2020).

### Practical Implications

The findings from this study have a range of practical implications for people involved in supporting Olympic athlete wellbeing. First, a process of normalization appeared to occur during the POP as athletes transitioned back to normality following a very exciting and stressful Olympic campaign. While some athletes described initial disappointment with their performance (lowered perceived self-worth/perception), over time they overcome these negative feelings through self-reflection, psychosocial support from family, friends, or psychologists, or planned return to work/study/competition. Even in situations where athletes experienced success and adequate support was available, there was an immediate adjustment period to navigate when returning home. The process of adaptation can be further compounded for athletes retiring from sport as the reality of feeling disconnected from teammates, coaches, and friends sets in soon after competition finishes. It is therefore not surprising that many athletes in this study perceived the POP as a challenging event that lowered subjective wellbeing until re-adaptation occurred (for some athletes, up to 2 years after the 2016 Games). While it still remains unclear is how long this process of normalization might take, athletes, coaches, and sporting organizations need to be conscious of the challenges that athletes encounter when transitioning back to normality following an Olympic campaign, and support athletes by using a variety of mechanisms to facilitate a return to ‘normal’ levels of wellbeing over time.

The many individual differences in athlete responses to performance outcomes indicates that maintaining realistic performance expectations is essential. Athletes should maintain a broad focus about the impact of being an Olympic athlete by balancing sport and life goals and focusing on internal measures of success. These expectations should be matched by coach, NSO, and AOC goals to minimize the pressure of external performance indicators and maintain wellbeing during the POP. Following an Olympic Games, support from family and friends was critical to athlete wellbeing and it was clear that athletes benefited from making post-Olympic plans *prior to* an Olympic campaign (e.g., a holiday, return to work, study, or professional competitions). Assisting athletes with the planning process early in the quadrennial cycle is likely to have a positive impact on post-Games wellbeing.

A higher-level oversight of athlete mental health and wellbeing would be valuable (beyond individuals and their teams) because while some athletes felt supported, others seemed bitter and unsupported by their sport. NSOs should ensure a range of personalized support is available due to stigma associated with reaching out for mental health challenges but also because athletes may prefer to connect internally (welfare manager or sport psychologist) or externally (independent psychologist or other mental health professional) when seeking support during the POP. The newly developed Mental Health Unit - implemented following the POP described here - may assist in this goal in future Olympic cycles (Rice et al., 2020). Through this unit, sport governing bodies could standardize wellbeing support across sports—perhaps in the form of an athlete wellbeing employee—as it seemed important to have a central person to follow up athletes over an extended period during the POP (at least 12 months). Relatedly, more assistance with developing career options for life after sport (mentors within the sport/Olympic context and networks outside of sport) would be of benefit, particularly for athletes considering retirement.

## Conclusion

Overall, this study explored the experiences of Australian Rio Olympians to better understand what athletes encountered, and how this influenced their wellbeing, when navigating the POP. Drawing on the voices of 18 athletes enabled us to conclude that: (1) Olympic performance appraisal strongly influenced athletes’ well-being during the POP; (2) Planning pre-Games for the POP was a major coping resource in the transition to normality; and (3) Available support in the POP was important but more was desired. It is hoped that the findings will be useful for NSOs, coaches, athletes, and their families in formulating plans for to enhance athlete wellbeing during future Olympic campaigns.

## Data Availability Statement

The datasets generated for this study are not readily available because to protect anonymity of participants, data cannot be made available. Please contact the lead author for any further information. Requests to access the datasets should be directed to AB.

## Ethics Statement

The studies involving human participants were reviewed and approved by Western Sydney University Human Ethics Committee. The participants provided their written informed consent to participate in this study. Written informed consent was obtained from the individual(s) for the publication of any potentially identifiable images or data included in this article.

## Author Contributions

AB conceptualized the study, recruited participants, conducted interviews, supervised the analysis, and drafted the final manuscript. CW completed the literature review and data analysis while developing initial drafts of the manuscript. DO assisted with study conceptualization, revisions of data analysis, and manuscript drafts. LF completed interviews and data analysis as well as contributed to initial drafts of the manuscript. TH conceptualized the study, contributed to recruitment of participants, and assisted with drafts of the manuscript. All authors reviewed and approved the final manuscript.

## Conflict of Interest

The authors declare that the research was conducted in the absence of any commercial or financial relationships that could be construed as a potential conflict of interest.

## Footnotes

1 The AOC provided funding for this research directly to the lead author’s university.

2 Please see Supplementary Materials for the interview guide.
