# Supplementary material for: Exploring the Experiences and Well-Being of Australian Rio Olympians During the Post-Olympic Phase: A Qualitative Study
Source: Front Psychol. 2021 May 26;12:685322. doi: 10.3389/fpsyg.2021.685322 (PMC8187857; doi:10.3389/fpsyg.2021.685322)
Supplement: Supplementary file 1 [file Data_Sheet_1.docx]

Supplementary Material

# Interview Guide – Post-Games Experiences

Opening Questions – Involvement in sport

1. Tell us a little about your life growing up and initial experiences with sport. How did you get involved in sport?
2. How long have you been competing (in any sports)?
3. What prompted your decision to become an Olympic Athlete and when did this occur?

Pre and During Games Experience

1. Tell me about the lead up to the Rio Games. When did you find out you had qualified?
2. Let’s move onto the time during the Rio Olympic Games. Can you please tell me about your experiences during the Rio Olympics?

Post-Olympic Games Experiences

For the next set of questions, I would like you to answer all of the questions solely based on your experiences during the Post-Olympic Games timeframe:

1. What happened immediately following your event being completed?
2. Tell us about some of the people that supported you throughout your post-Olympic Games experiences.
3. Tell us about some of the things, if any, that made life difficult during the post-Olympic period.
4. Tell us a little about your decision to (a) continue on with training for the next Olympic Games OR (b) transition into retirement from Olympic Games participation.

Concluding Questions

1. What, if any, recommendations do you have for future Olympians, current Olympians, coaches, families, and administrators to help improve peoples’ experiences during the post-Olympic time period?

Are there any other comments you wish to add? Do you have any final questions or comments?

**THANK YOU FOR YOUR TIME.**
